# Bibliometric Analysis of Context, Trends, and Contents of Digital Health Technology Used in Dental Health

**DOI:** 10.1155/2023/5539470

**Published:** 2023-10-25

**Authors:** Chaitanya S. Buddhikot, Vikram Garcha, Vittaldas Shetty, Kadambari Ambildhok, Vineet Vinay, Utkarsha Deshpande, Dian Agustin Wahjuningrum, Alexander Maniangat Luke, Mohmed Isaqali Karobari, Ajinkya M. Pawar

**Affiliations:** ^1^Department of Public Health Dentistry, Sinhgad Dental College and Hospital, Sinhgad Rd, Pune, Maharashtra 411041, India; ^2^Department of Conservative Dentistry, Faculty of Dental Medicine, Universitas Airlangga, Surabaya City, East Java 60132, Indonesia; ^3^Department of Clinical Sciences, College of Dentistry, Ajman University, Ajman, UAE; ^4^Center for Medical and Bio-Allied Health Sciences Research (CMBAHSR), Ajman University, Ajman, UAE; ^5^Department of Restorative Dentistry & Endodontics, Faculty of Dentistry, University of Puthisastra, Phnom Penh 12211, Cambodia; ^6^Department of Conservative Dentistry & Endodontics, Saveetha Institute of Medical and Technical Sciences University, Chennai, 600077 Tamil Nadu, India; ^7^Department of Conservative Dentistry and Endodontics, Nair Hospital Dental College, Mumbai, 400008 Maharashtra, India

## Abstract

Digital tools and apps are revolutionizing healthcare and provide creative answers to urgent problems. Through teamwork and the incorporation of digital technologies, dentistry has experienced a remarkable revolution. A large body of scholarly research backs up this trend. The context, trends, and content of digital health technology in oral and dental health are examined in our bibliometric analysis. Using targeted keywords and synonyms, an organized searching technique was used in the Scopus database, yielding 1942 articles that were extracted into a CSV file. To acquire insights into the content, trends, and context, visualization using VOSviewer 1.6.18 and a variety of analyses—including coauthorship, citation, cooccurrence of author keywords, bibliographic coupling, and cocitation—were executed. The analysis revealed that the USA and the UK contributed to a significant quantity of the literature, with newer contributions coming from nations like India. Cone Beam Computed Tomography, Dental Caries, and Artificial Intelligence were prominent keywords. It is important to note that BMC Oral Health was associated with a sizable number of the papers. This bibliometric analysis provides insightful information about the context, content, and trends of digital health in the field of oral and dental health. By implementing the right technology, policymakers can use this information to increase oral health, encourage dental literacy, and improve access to dental treatment. It is vital to take into account the wide variety of technologies and their classifications based on dental services and contextual variables.

## 1. Introduction

Dentistry is not immune to the pervasive buzzword of digitization that permeates various industries in today's business landscape [1, 2]. The limitations and challenges that were once prevalent in clinical and technical procedures just a few years ago have been effectively tackled thanks to the relentless progress in information technology (IT) [3, 4]. Moreover, the societal and cultural norms of advanced nations have undergone significant transformations, aligning with and bolstering the digitalization trend. These shifts include urbanization, centralized systems, increased mobility, and the omnipresence of smartphones and tablets, coupled with the interconnectedness of the Internet of things (IoT), as well as marketplaces driven by efficiency [5, 6].

Through the utilization of digital tools and applications, healthcare professionals can explore novel approaches to address pressing challenges such as the heightened vulnerability of the aging population to chronic ailments and the escalating costs associated with lifelong healthcare expenses [7, 8]. Within the realm of dental care, numerous digital procedures for manufacturing and processing have already been integrated into treatment protocols, notably within the rapidly expanding domains of rapid prototyping (RP) and computer-aided design/manufacturing (CAD/CAM) [9].

The advent of artificial intelligence (AI) and machine learning (ML) has ushered in a realm of possibilities for automated processing within the realm of radiological imaging. Building upon this technological foundation, the creation of virtual dental patients by superimposing diverse imaging data and conducting noninvasive simulations to compare various outcomes prior to any clinical intervention has been further augmented by the integration of augmented reality and virtual reality (AR/VR) technologies. These groundbreaking advancements have been made viable due to the exponential growth in computing power, yet their full potential applications are still on the horizon [10]. While the digital landscape continues to be explored, the remarkable benefits it holds are not yet fully recognized. It is imperative to shift our focus beyond industry-oriented studies and redirect our attention towards patient-centered outcomes, encompassing basic scientific research, clinical trials, and the valuable information they yield, which can ultimately lead to the development of innovative therapeutic approaches [11].

In recent trends, the field of dentistry has witnessed remarkable advancements in digital radiography, offering significant advantages such as time-saving during treatments, convenient storage, and effortless data transfer [12]. This technology has greatly enhanced efficiency by reducing the time required for data retrieval and analysis [13]. Moreover, patients' acceptance and understanding of dental procedures have been elevated through the utilization of live videos, three-dimensional animations, voice-activated software, and intraoral cameras9. Over the past decade, CAD/CAM technology has empowered dental practitioners to shift some restoration manufacturing processes to chairside operations exclusively [14]. This transformation not only has enhanced the accuracy and effectiveness of treatments [13] but also has resulted in improved work efficiency while reducing time and cost for patients. A study conducted by Joda and Brägger [15] highlighted the superior efficiency and effectiveness of digital workflows compared to established conventional pathways, offering a more cost-effective treatment option for patients. Furthermore, a controlled clinical trial conducted by Yuzbasioglu et al. [16] concluded that digital techniques for creating impressions not only saved time but also were preferred by patients compared to conventional methods. Additionally, the overall procedure time was shorter for digital techniques, and patients reported higher levels of comfort, even when performed by experienced operators using conventional methods.

Various studies are being conducted with the goal of comparing the effects of various digital technology-based therapies on dental health. For instance, Zolfaghari et al. [17] reported that the examined moms' knowledge and practice of oral health increased after a month of using a straightforward app without gamification and its gamified version. Wallace et al. [18] showed that after introducing a telephone consultation for kids and their parents, both the quantity of pointless in-person consultations and the length of waiting lists were reduced. The use of teledentistry in a children's hospital system pilot has also been shown by Hammersmith et al. [19] with favorable results for both careers and dentists.

The profound influence of technology on oral and dental health underscores the importance of comprehending the specific contexts and contents addressed within the current research. In essence, despite the notable impact of digital health technology on oral health, there remains uncertainty regarding the extent to which studies have been conducted to investigate the specific settings and contents related to this subject matter. Hence, in order to investigate the context, content, and trends of digital health technology used in dental health, this bibliometric analysis was conducted.

## 2. Materials and Methods

A search strategy was formulated using the synonyms for context, trends, and contents of digital and dental and oral health. The search strategy was used in Scopus database, and the search strategy employed is shown in Table 1. Article retrieval was done in CSV format. All the articles were segregated and if duplicates were present were systematically removed. A final of 1942 articles were subjected to visualization using VOSviewer version 1.6.18, and coauthor analysis, cooccurrence of keywords, citation analysis, and bibliographic coupling were done on the obtained data.

“Citation analysis” is predicated on the premise that “citations” serve as indicators of “intellectual connections” established between publications, whereby one publication references another. This technique is aimed at ascertaining the influence of a publication by evaluating the quantity of citations it garners. The methodology of “cocitation analysis” is predicated on the assumption that publications which are often referenced together exhibit thematic similarity. One advantage associated with the utilization of “cocitation analysis” is that it enables business researchers to not only identify highly significant publications but also uncover theme clusters within the field.

The coword analysis, also known as “cooccurrence” analysis, is based on the assumption that words that regularly cooccur in a given context are likely to have a thematic link with each other. The analysis of “coauthorship” investigates the dynamics and relationships between researchers within a certain research domain.

## 3. Results

### 3.1. Coauthorship Analysis

#### 3.1.1. Authors

**Figure 1:**
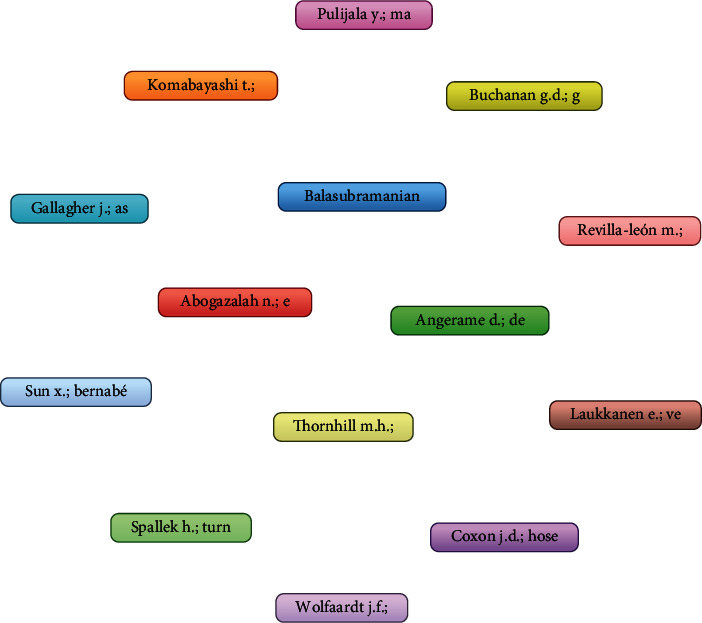
Authorship analysis.

When the coauthorship analysis was done keeping the unit of analysis as authors and setting the threshold of minimum 2 number of documents of an author, it was found that 1930 authors were present; however, only 14 met the threshold level. The total link strength was established between 14 authors wherein it was found that for 2 documents, there were authors who obtained a citation of 158. However, it was observed that there was no link strength between these authors (Table 2 and Figure 1).

#### 3.1.2. Organization

**Figure 2:**
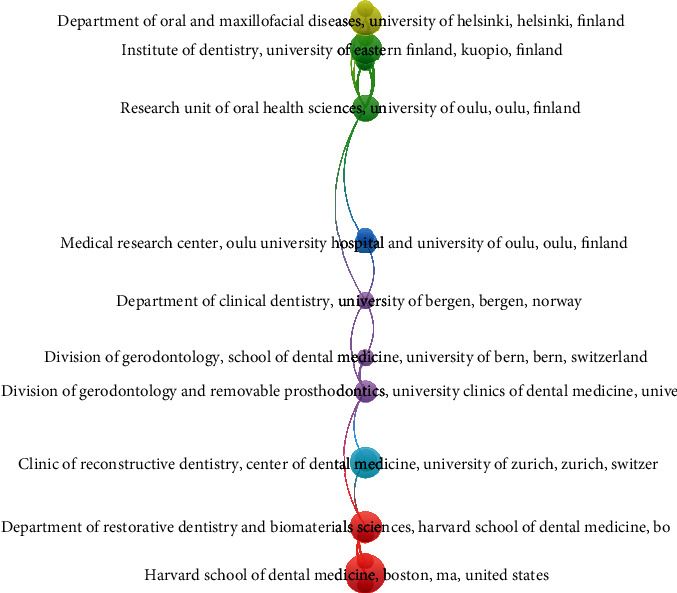
Coauthorship analysis for organization.

When coauthorship analysis was done keeping unit of analysis as organization, it was observed that out of 6093 organizations meeting the threshold of minimum of 2 documents and minimum of 2 citations per document, it was found that there were 306 universities that were meeting the threshold limit. It was observed that the Department of Dental Medicine, Karolinska Institutet, Stockholm, Sweden, had a maximum of 12 documents having 99 citations; however, the Department of Periodontics and Oral Medicine, University of Michigan, had 10 documents receiving 770 citations, while the link strength appeared to be highest for School of Dental Sciences, Newcastle University, UK, where a highest of 14 link strengths was obtained. Even though there are 306 universities that met the threshold, it was observed that only 20 of them had connected items for coauthorship and the analysis of the same was carried out of 20 items (Table 3 and Figure 2).

#### 3.1.3. Countries

**Figure 3:**
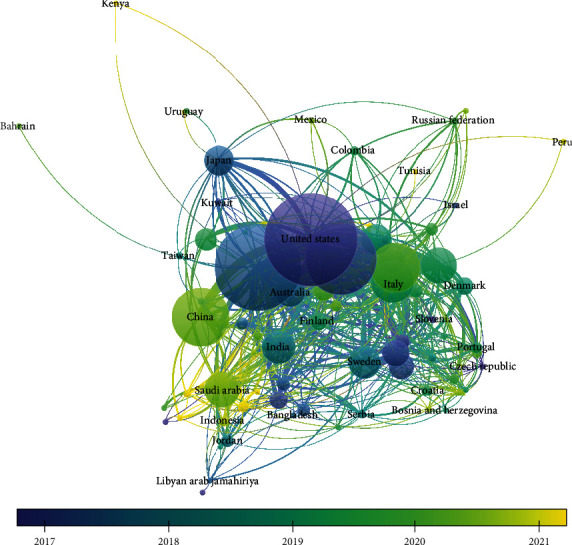
Coauthorship analysis for country.

The coauthorship analysis done for the countries revealed that of the 125 countries, only 84 met the threshold of minimum of 2 documents per country and minimum of 2 citations per country. The largest connected data set consisted of 84 countries; of these 84 countries studied, it was found that the highest documents and citation were observed for the USA with 353 documents and the USA had obtained 9468 citations, respectively. Of the 84 countries, only 81 presented largest linked item. The overlay visualization portrays that the largest publication and citation were obtained by the US followed by the UK followed by Australia, Switzerland, South Korea, Saudi Arabia, Sweden, India, etc. While these countries have higher number of publication period of 2017-2020, countries like Indonesia, Kenya, Yemen and Vietnam have been publishing in recent time period (Table 4 and Figure 3).

### 3.2. Cooccurrence

#### 3.2.1. Author Keywords

**Figure 4:**
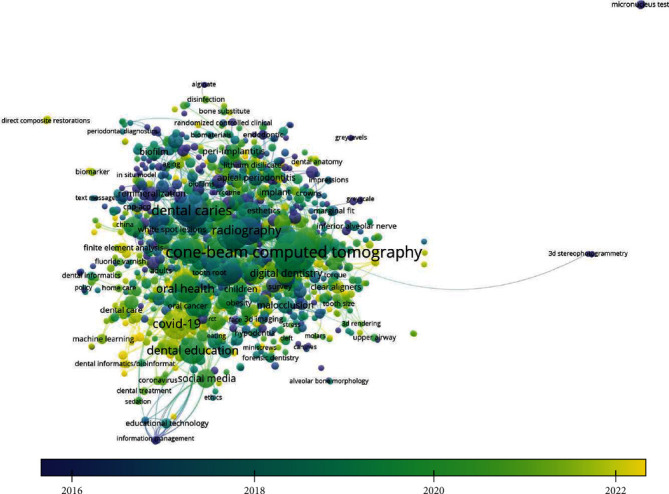
Cooccurrence of author keywords with citations and total link strength (overlay visualization).

Keeping the minimum threshold of 2 keywords, it was found that there were 4225 keywords of which 1009 met the threshold limit of which there were more than 60 truncated words which were repeated after appropriate removal; it was found that the final set consisted of 941 items having link strength between them. The overlay visualization depicted that the highest occurring keywords were Cone Beam Computed Tomography followed by Orthodontics followed by Dental Caries; however, it was found that in recent era, a much interest in deep learning and machine learning was developed and a new concept generation acting on primary health care is established. Machine Learning CNN (Convoluted Neural Network) has also seemed to be used as author keywords (Table 5 and Figure 4).

### 3.3. Citation Analysis

#### 3.3.1. Documents

Of the 1942 documents studied, it was found that only 1407 met the threshold of having minimum of 2 citations. The highest citation was obtained by Ludlow et al. [20] wherein the document received the highest citation of 590. However, there were no links present between these documents.

#### 3.3.2. Sources

**Figure 5:**
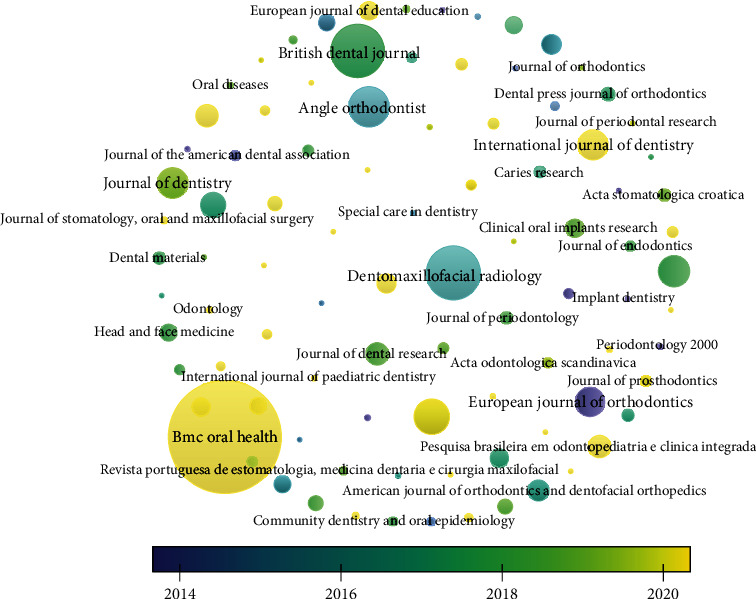
Overlay visualization of analysis of citation for sources.

**Figure 6:**
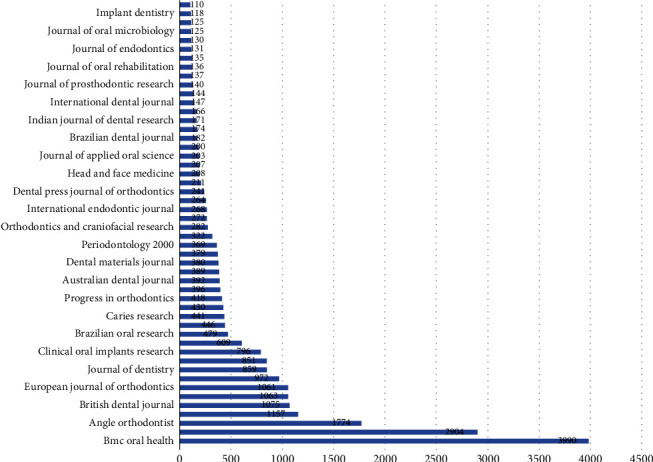
Sources with more than 100 citations.

The citation analysis for source unit revealed that for a minimum of 2 documents and 2 citations per source out of 109 sources, 93 met the threshold. The analysis revealed that BMC Oral Health had obtained the highest citation for 339 published documents followed by Dentomaxillofacial Radiology journal which had received 2904 citations for 119 documents published; however, it was also observed that there were no link strengths present between them; it was found that BMC Oral Health has obtained these higher citations post 2020 (Figures 5 and 6).

### 3.4. Sources

#### 3.4.1. Authors

There were 1930 authors of which only 14 met the threshold of having 2 documents with 2 citations; it was elaborated that Ayoub and Pulijala [21] had the highest citation of 158; however, there was again no link strength between authors (Table 6).

#### 3.4.2. Organizations

**Figure 7:**
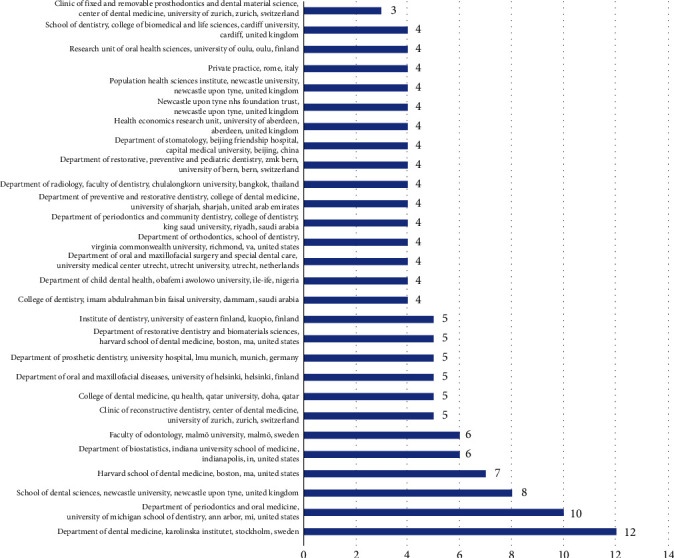
Documents published by organizations.

There were 6930 organizations present of which 306 met the threshold of minimum of 2 documents and 2 citations; of these organizations, it was found that the highest citation was obtained by the Department of Periodontics and Oral Medicine, University of Michigan School of Dentistry, USA (770), followed by the University of Varese (327) (Figure 7).

#### 3.4.3. Countries

**Figure 8:**
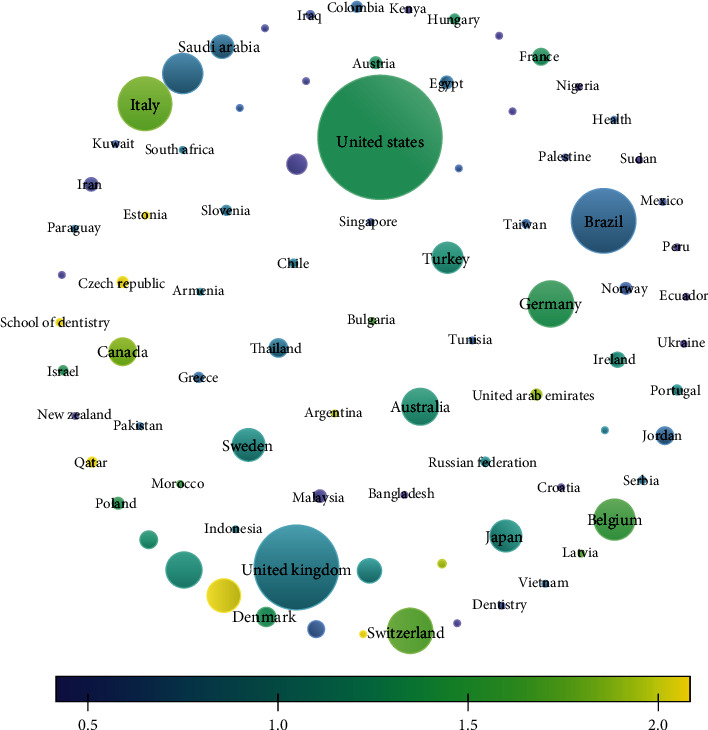
Overlay visualization of citation for countries.

Citation analysis for countries portrayed that of the 125 countries, 84 met the threshold of having citations. The trend depicted that the USA had the most citation of 9468 followed by the UK (4982) whereas India was observed to be having citation of 468 for 88 documents published. It was observed that in recent time, it was the UAE, Egypt, Qatar, and Nepal that published more literature (Figure 8).

### 3.5. Bibliographic Coupling

#### 3.5.1. Documents

**Figure 9:**
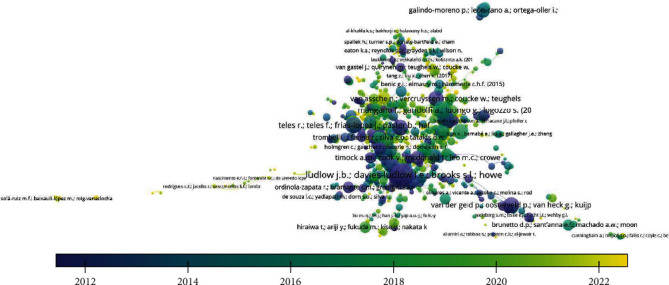
Overlay visualization of bibliographic coupling for documents.

Of the 1942 documents assessed for the bibliometric coupling, it was found that only 1407 met the threshold limit of minimum of 2 citations per document. It was observed that Ludlow et al. [20] had the highest citation of 590 whereas Revilla-León et al. [22] had the highest link strength with 111 documents. However, only 1025 of the 1047 data were depicting link strength. The overlay visualization depicted that Bayraktar and Ayan [23] who published a study on diagnosis of interproximal caries lesion with deep CNN in digital bitewing radiographs was receiving a high bibliographic coupling in recent time, and similar finding was observed for Kühnisch et al. [24] who studied caries detection on intraoral images using AI (Figure 9).

#### 3.5.2. Sources

**Figure 10:**
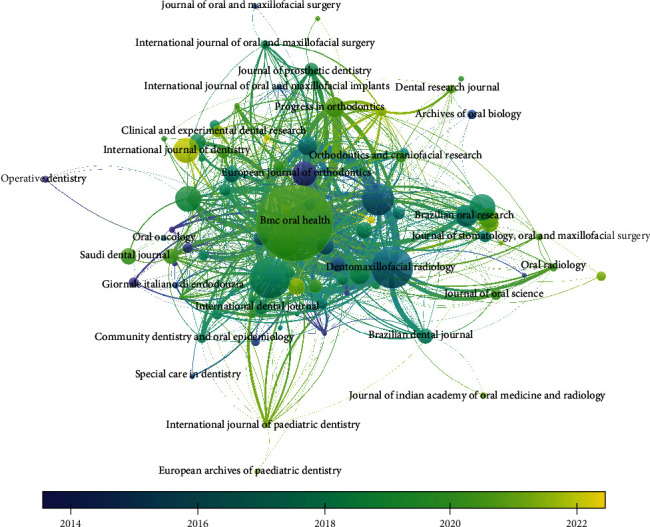
Overlay visualization of bibliographic coupling for sources.

The bibliographic coupling for sources revealed that for 109 sources, only 93 met the threshold of 2 documents and 2 citations of a source; it was observed that BMC Oral Health journal had maximum documents with 3990 citations and 1637 total link strengths, followed by Dentomaxillofacial Radiology with 119 documents and 2904 citations with 1110 total link strengths followed by British Dental Journal with 116 documents and 1075 citations with 487 link strengths; however, of these 93 studies, only 91 studies identified having connected items between them. The overlay visualization depicted that International Journal of Dentistry, Clinical and Experimental Research, and Odontology have bibliographic coupling occurring post 2020; it was observed that even though the highest bibliographic coupling was present in BMC Oral Health and Dentomaxillofacial Radiology journal, these couplings were obtained in 2014-2020 (Figure 10).

#### 3.5.3. Authors

Of the 1930 authors, only 14 authors met the threshold limit of 2 documents and 2 citations; however, it was observed that there was no link present between these 14 authors.

#### 3.5.4. Countries

The bibliographic coupling for countries revealed that of the 125 countries, only 84 met the threshold limit. The highest bibliographic coupling was for the USA with 9468 citations followed by the UK with 320 documents and 4982 citations; it was found that India had 83 documents with 468 citations having a total link strength of 3029. The overlay visualization depicted that the bibliographic coupling was highest for the USA, the UK, and India; however, these couplings were obtained from 2017 to 2019 whereas in recent era, countries like Qatar, Nepal, and Vietnam obtained bibliographic coupling (Table 7).

### 3.6. Cocitation Analysis

#### 3.6.1. Cited References

Of the 66238 references, it was found that 3421 met the threshold of minimum of 2 cited references. However, it was also observed that only 3333 had the largest connected sources. The highest cocitation for references was obtained by Landis and Koch [25].

#### 3.6.2. Cited Authors

**Figure 11:**
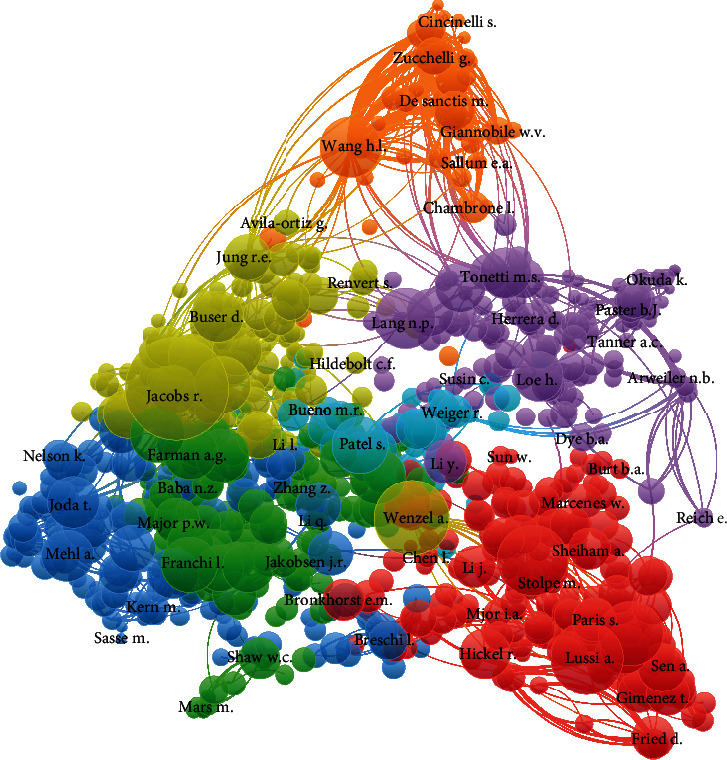
Visualization of cocitations (cited authors).

Cocitations between cited authors were present for 31219 of 95068 having a minimum of 2 citations; it was observed that authors Jacob R and Wenzel had the highest citation for cocited authors whereas the least cocited author was Moradian-Oldak J (Figure 11).

## 4. Discussion

In the present study, a total of 1942 literature works have been studied. All the literature assessed belonged to English language which were purely published in open access journals.

Our study has hence provided insights into the usage of digital health technologies in dental and oral healthcare. We observed a significant increase in the adoption of digital technologies commencing from 2005, as reflected in the index keywords. These technologies included computer-assisted therapy/surgery, computer simulation, computer programs, image processing, nuclear magnetic resonance (NMR) imaging, and audio-visual equipment. Additionally, telemedicine, mobile applications, virtual reality, and medical information were commonly associated with terms like dental caries and dental procedures. Furthermore, we noticed a notable rise in publications, particularly from 2015 onwards, with the majority of articles published in 2021. This trend suggests a parallel occurrence of the COVID-19 pandemic and the development of teledentistry. An interesting finding was the cooccurrence of keywords such as Cone Beam Computed Tomography, Orthodontics, and Dental Caries. However, we also observed a recent surge of interest in deep learning and machine learning, indicating the emergence of innovative concepts that impact primary healthcare. Additionally, our study revealed a gap in the implementation of newer digital technologies, such as IoT, artificial intelligence, and gamification (online gaming, video games, etc.), in the field of oral and dental health.

In the present literature of our bibliometric analysis, it was found that the coauthorship between organizations has revealed that the Department of Dental Medicine, Karolinska Institutet, Stockholm, Sweden, and the Department of Periodontics and Oral Medicine, University of Michigan, followed by the School of Dental Sciences, Newcastle University, UK, were the top leading institutes with the highest coauthorship. A similar reflection of the finding was noted with the countries that the USA followed by the UK depicted a higher coauthorship, cocitation, and citation. The main reason behind this is a higher number of publishing houses belonging to American nations followed by European nations while due to availability of research and grants, it is easy to perform and conduct multiple researches in this soil. Our finding is in consensus with bibliometric analysis conducted by Bastani et al. [26], which elicited that the highest publication was observed for the United Kingdom followed by the United States of America whereas in bibliometric analysis presented by van der Wouden et al. [27], a yet again similar trend was observed wherein a higher amount of literature has been published by the United States of America. As per our studied trend of publication from 1985 to 2015, we still sought a similar finding. Our study also revealed that the most recent publication is occurring from countries like Yemen, Ecuador, and India where a plethora of research has just initiated.

Strengths were not depicted in the overlay visualization.

With respect to context, it is explored from the visualization that a higher number of research publication include keywords of CBCT and Caries Diagnosis. Due to the emergence of artificial intelligence, many authors have started to conduct, develop, and publish their research work related to the field. The study published in 2021 includes AI as important keywords wherein it was found that Mertens et al. [28] conducted a study that used AI-based diagnostic support for proximal caries detection whereas Cantu et al. [29] published studies which included caries, AI, and Bitewing Radiographs as cooccurring keywords while their study is aimed at applying deep learning to detect caries lesions of different radiographic extension on bitewings, hypothesizing it to be significantly more accurate than individual dentists. Similarly, an explicit number of research was published having CBCT as keywords as well.

An interesting finding revealed from our bibliometric analysis was that the highest source of articles having coauthorship, cocitation, and citation was published in BMC Oral Health. This journal published the work related to prevention, diagnosis, and management of disorders of the mouth, teeth, and gums, as well as related molecular genetics, pathophysiology, and epidemiology. The journal promises 42 days to first decision for all manuscripts (median) and 56 days to first decision for reviewed manuscripts only (median) while having an impact factor of 3.748 (2-year impact factor) (2021) and 3.917 (5-year impact factor) (2021). Open access journal with substantial publication charges a higher number of articles which were observed to be published in this journal.

Our results have portrayed the context, content, and trends of published literature regarding digital health and oral and dental health. Our limitations included only single database which was used for analysing and visualizing our objective, while our key strengths include the usage of the most holistic software (VOSviewer) developed for visualization of our objective.

## 5. Conclusions

This bibliometric study showcases the increasing adoption of digital technology in oral and dental health, presenting policymakers with a unique chance to enhance access, literacy, knowledge, and services. Nevertheless, it is essential to acknowledge that technology types and classifications differ over time and across various dental areas. Through the utilization of insights derived from this study, decision-makers acquire valuable knowledge regarding technology trends, types, applications, benefits, and limitations in the realm of oral health. This knowledge equips them to make informed decisions when implementing efficient technologies, thereby improving access and ultimately enhancing oral health outcomes within specific contexts.

## Figures and Tables

**Table 1:** Sources and searched strategy information.

Database	Searched strategies
Scopus	(ALL (contents) OR ALL (constituents)) OR trends OR shift OR course OR drift OR tendency OR context OR factors OR circumstances OR condition AND digital AND dental AND health OR digital AND oral AND health AND (LIMIT-TO (DOCTYPE, “ar”) OR LIMIT-TO (DOCTYPE, “cp”) OR LIMIT-TO (DOCTYPE, “re”)) AND (LIMIT-TO (SUBJAREA, “MEDI”)) AND (LIMIT-TO (LANGUAGE, “English”)) AND (LIMIT-TO (EXACTSRCTITLE, “International Journal Of Environmental Research And Public Health”) OR LIMIT-TO (EXACTSRCTITLE, “Dentomaxillofacial Radiology”) OR LIMIT-TO (EXACTSRCTITLE, “Journal Of Clinical Medicine”) OR LIMIT-TO (EXACTSRCTITLE, “Journal Of Biological Regulators And Homeostatic Agents”) OR LIMIT-TO (EXACTSRCTITLE, “Oral Surgery Oral Medicine Oral Pathology And Oral Radiology”) OR LIMIT-TO (EXACTSRCTITLE, “Journal Of Oral And Maxillofacial Surgery”) OR LIMIT-TO (EXACTSRCTITLE, “Oral Diseases”) OR LIMIT-TO (EXACTSRCTITLE, “Pesquisa Brasileira Em Odontopediatria E Clinica Integrada”) OR LIMIT-TO (EXACTSRCTITLE, “Imaging Science In Dentistry”) OR LIMIT-TO (EXACTSRCTITLE, “Archives Of Oral Biology”) OR LIMIT-TO (EXACTSRCTITLE, “Oral Radiology”) OR LIMIT-TO (EXACTSRCTITLE, “Photodiagnosis And Photodynamic Therapy”) OR LIMIT-TO (EXACTSRCTITLE, “Cochrane Database Of Systematic Reviews”) OR LIMIT-TO (EXACTSRCTITLE, “International Journal Of Oral And Maxillofacial Surgery”) OR LIMIT-TO (EXACTSRCTITLE, “Community Dentistry And Oral Epidemiology”) OR LIMIT-TO (EXACTSRCTITLE, “Journal Of Medical Internet Research”) OR LIMIT-TO (EXACTSRCTITLE, “Oral Surgery Oral Medicine Oral Pathology Oral Radiology And Endodontology”) OR LIMIT-TO (EXACTSRCTITLE, “Journal Of Indian Academy Of Oral Medicine And Radiology”) OR LIMIT-TO (EXACTSRCTITLE, “British Journal Of Oral And Maxillofacial Surgery”) OR LIMIT-TO (EXACTSRCTITLE, “Community Dental Health”) OR LIMIT-TO (EXACTSRCTITLE, “Frontiers In Bioengineering And Biotechnology”) OR LIMIT-TO (EXACTSRCTITLE, “Anatomical Sciences Education”) OR LIMIT-TO (EXACTSRCTITLE, “Cancers”) OR LIMIT-TO (EXACTSRCTITLE, “Indian Journal Of Public Health Research And Development”) OR LIMIT-TO (EXACTSRCTITLE, “Forensic Science International”) OR LIMIT-TO (EXACTSRCTITLE, “Journal Of Healthcare Engineering”) OR LIMIT-TO (EXACTSRCTITLE, “Journal Of Stomatology Oral And Maxillofacial Surgery”) OR LIMIT-TO (EXACTSRCTITLE, “BMJ Open”) OR LIMIT-TO (EXACTSRCTITLE, “Head And Face Medicine”) OR LIMIT-TO (EXACTSRCTITLE, “Children”) OR LIMIT-TO (EXACTSRCTITLE, “Lasers In Medical Science”) OR LIMIT-TO (EXACTSRCTITLE, “Frontiers In Cellular And Infection Microbiology”) OR LIMIT-TO (EXACTSRCTITLE, “Medicina Oral Patologia Oral Y Cirugia Bucal”) OR LIMIT-TO (EXACTSRCTITLE, “Radiation Protection Dosimetry”) OR LIMIT-TO (EXACTSRCTITLE, “Gerodontology”) OR LIMIT-TO (EXACTSRCTITLE, “Journal Of Clinical Pediatric Dentistry”) OR LIMIT-TO (EXACTSRCTITLE, “Journal Of Oral Biology And Craniofacial Research”) OR LIMIT-TO (EXACTSRCTITLE, “Open Access Macedonian Journal Of Medical Sciences”) OR LIMIT-TO (EXACTSRCTITLE, “Oral Surgery Oral Medicine Oral Pathology Oral Radiology And Endodontics”) OR LIMIT-TO (EXACTSRCTITLE, “Journal Of Public Health Dentistry”) OR LIMIT-TO (EXACTSRCTITLE, “Medicine United States”) OR LIMIT-TO (EXACTSRCTITLE, “Oral Surgery”) OR LIMIT-TO (EXACTSRCTITLE, “Frontiers In Public Health”) OR LIMIT-TO (EXACTSRCTITLE, “Journal Of Forensic Odonto Stomatology”) OR LIMIT-TO (EXACTSRCTITLE, “Frontiers In Medicine”) OR LIMIT-TO (EXACTSRCTITLE, “International Journal Of Clinical Pediatric Dentistry”) OR LIMIT-TO (EXACTSRCTITLE, “Journal Of Forensic And Legal Medicine”) OR LIMIT-TO (EXACTSRCTITLE, “Journal Of Maxillofacial And Oral Surgery”) OR LIMIT-TO (EXACTSRCTITLE, “Medical Science Monitor”) OR LIMIT-TO (EXACTSRCTITLE, “Oral Surgery Oral Medicine Oral Pathology”) OR LIMIT-TO (EXACTSRCTITLE, “Pakistan Journal Of Medical And Health Sciences”) OR LIMIT-TO (EXACTSRCTITLE, “Biomedicines”) OR LIMIT-TO (EXACTSRCTITLE, “Clinical Anatomy”) OR LIMIT-TO (EXACTSRCTITLE, “Frontiers In Physiology”) OR LIMIT-TO (EXACTSRCTITLE, “Journal Of Digital Imaging”) OR LIMIT-TO (EXACTSRCTITLE, “Journal Of Orthodontics”) OR LIMIT-TO (EXACTSRCTITLE, “Minerva Stomatologica”) OR LIMIT-TO (EXACTSRCTITLE, “Oral And Maxillofacial Surgery Clinics Of North America”) OR LIMIT-TO (EXACTSRCTITLE, “Journal Of Oral Microbiology”) OR LIMIT-TO (EXACTSRCTITLE, “Photobiomodulation Photomedicine And Laser Surgery”) OR LIMIT-TO (EXACTSRCTITLE, “Telemedicine And E Health”) OR LIMIT-TO (EXACTSRCTITLE, “British Journal Of Radiology”) OR LIMIT-TO (EXACTSRCTITLE, “Journal Of Medical Imaging”) OR LIMIT-TO (EXACTSRCTITLE, “Lasers In Surgery And Medicine”) OR LIMIT-TO (EXACTSRCTITLE, “Photomedicine And Laser Surgery”) OR LIMIT-TO (EXACTSRCTITLE, “Prosthesis”) OR LIMIT-TO (EXACTSRCTITLE, “Sleep And Breathing”) OR LIMIT-TO (EXACTSRCTITLE, “Studies In Health Technology And Informatics”) OR LIMIT-TO (EXACTSRCTITLE, “Surgical And Radiologic Anatomy”) OR LIMIT-TO (EXACTSRCTITLE, “BMC Health Services Research”) OR LIMIT-TO (EXACTSRCTITLE, “Cranio Journal Of Craniomandibular And Sleep Practice”) OR LIMIT-TO (EXACTSRCTITLE, “European Journal Of Orthodontics”) OR LIMIT-TO (EXACTSRCTITLE, “European Radiology”) OR LIMIT-TO (EXACTSRCTITLE, “International Journal Of Medical Informatics”) OR LIMIT-TO (EXACTSRCTITLE, “International Journal Of Periodontics And Restorative Dentistry”) OR LIMIT-TO (EXACTSRCTITLE, “Journal Of Biomechanics”) OR LIMIT-TO (EXACTSRCTITLE, “Journal Of Bone And Mineral Research”) OR LIMIT-TO (EXACTSRCTITLE, “Minerva Dental And Oral Science”) OR LIMIT-TO (EXACTSRCTITLE, “Dental And Medical Problems”) OR LIMIT-TO (EXACTSRCTITLE, “Journal Of Education And Health Promotion”) OR LIMIT-TO (EXACTSRCTITLE, “Journal Of The College Of Physicians And Surgeons Pakistan”) OR LIMIT-TO (EXACTSRCTITLE, “Journal Of The Korean Association Of Oral And Maxillofacial Surgeons”)) AND (LIMIT-TO (SUBJAREA, “DENT”) OR LIMIT-TO (SUBJAREA, “HEAL”))

**Table 2:** Coauthorship analysis for authors.

Sr. no.	Author	Citations
1	Pulijala Y.; Ma M.; Pears M.; Peebles D.; Ayoub A.	158
2	Revilla-León M.; Jiang P.; Sadeghpour M.; Piedra-Cascón W.; Zandinejad A.; Özcan M.; Krishnamurthy V.R.	155
3	Thornhill M.H.; Dayer M.J.; Durkin M.J.; Lockhart P.B.; Baddour L.M.	63
4	Gallagher J.; Ashley P.; Petrie A.; Needleman I.	51
5	Spallek H.; Turner S.P.; Donate-Bartfield E.; Chambers D.; Mcandrew M.; Zarkowski P.; Karimbux N.	42
6	Abogazalah N.; Eckert G.J.; Ando M.	40
7	Sun X.; Bernabé E.; Liu X.; Gallagher J.E.; Zheng S.	29
8	Laukkanen E.; Vehkalahti M.M.; Kotiranta A.K.	25
9	Balasubramanian M.; Spencer A.J.; Short S.D.; Watkins K.; Chrisopoulos S.; Brennan D.S.	23
10	Coxon J.D.; Hosey M.-T.; Newton J.T.	12
11	Komabayashi T.; Ahn C.; Zhang S.; Zhu Q.; Spångberg L.S.W.	8
12	Buchanan G.D.; Gamieldien M.Y.; Fabris-Rotelli I.; Van Schoor A.; Uys A.	3
13	Wolfaardt J.F.; Brecht L.E.; Taft R.M.	3
14	Angerame D.; De Biasi M.; Franco V.; Bevilacqua L.; Castaldo A.	2

**Table 3:** Coauthorship analysis for organization depicting documents and citations.

Sr. no.	Organization	Documents	Citations
1	Department of Periodontics and Oral Medicine, University of Michigan School of Dentistry, Ann Arbor, MI, United States	10	770
2	University of Varese, Department of Medicine and Surgery, Dental School, Varese, Italy	2	327
3	Private practice, Bologna, Italy	2	318
4	Private practice, Dallas, TX, United States	3	184
5	Researcher, Revilla Research Center, Madrid, Spain	3	174
6	Department of Prosthodontics, Bauru School of Dentistry, University of São Paulo, Bauru, Brazil	2	164
7	Affiliate faculty graduate prosthodontics, University of Washington, Seattle, WA, United States	2	155
8	Assistant faculty mechanical engineering, Texas A&M University, College Station, TX, United States	2	155
9	Associate professor and program director AEGD residency, College of Dentistry, Texas A&M University, Dallas, TX, United States	2	155
10	Clinic of Fixed and Removable Prosthodontics and Dental Material Science, Center of Dental Medicine, University of Zurich, Zurich, Switzerland	3	155
11	Graduate research assistant, mechanical engineering, Texas A&M University, College Station, TX, United States	2	155
12	Professor and head, Dental Materials Unit, Center for Dental and Oral Medicine, University of Zürich, Zürich, Switzerland	2	155
13	Hamad Medical Corporation, Doha, Qatar	2	149
14	School of Dentistry, University of Washington, Seattle, WA, United States	2	141
15	College of Dental Medicine, QU Health, Qatar University, Doha, Qatar	5	140
16	Department of Radiology, Faculty of Dentistry, Chulalongkorn University, Bangkok, Thailand	4	132
17	School of Dental Sciences, Newcastle University, Newcastle upon Tyne, United Kingdom	8	120
18	National Institute for Health Research (NIHR) Biomedical Research Unit in Nutrition, Diet and Lifestyle at the University Hospitals Bristol NHS Foundation Trust, University of Bristol, Bristol, United Kingdom	2	117
19	School of Oral and Dental Sciences, University of Bristol, Bristol, United Kingdom	2	117
20	Department of Restorative Dentistry and Biomaterials Sciences, Harvard School of Dental Medicine, Boston, MA, United States	5	105
21	Department of Biostatistics, University of Washington, Seattle, WA, United States	2	101
22	Department of Dental Medicine, Karolinska Institutet, Stockholm, Sweden	12	99
23	Faculty of Dentistry, Chiang Mai University, Chiang Mai, Thailand	3	94
24	Department of Oral Health Sciences, KU Leuven and Dentistry, University Hospitals Leuven, Leuven, Belgium	2	90
25	Private practice, Rome, Italy	4	89
26	Department of Pathology, University of Texas, Health Science Center at San Antonio, San Antonio, TX, United States	2	88
27	School of Dentistry, University of Adelaide, Adelaide, SA 5005, Australia	2	84
28	UCL Eastman Dental Institute, WC1X 8LD, 256 Grays Inn Road, London, United Kingdom	3	84
29	Department of Biostatistics, Indiana University School of Medicine, Indianapolis, IN, United States	6	83
30	Department of Biomedical, Surgical and Dental Sciences, Foundation IRCCS CA' Granda Polyclinic, University of Milan, Milan, Italy	2	82
31	Top Institute Food and Nutrition, Wageningen, Netherlands	2	82
32	Department of Clinical Dentistry, University of Bergen, Bergen, Norway	2	81
33	Division of Gerodontology and Removable Prosthodontics, University Clinics of Dental Medicine, University of Geneva, Geneva, Switzerland	3	80
34	Division of Gerodontology, School of Dental Medicine, University of Bern, Bern, Switzerland	2	80
35	Department of Oral Diagnostics, Digital Health and Health Services Research, Charité - Universitätsmedizin Berlin, Germany	2	71
36	Department of Orthodontics, Seoul National University Dental Hospital, Seoul, South Korea	2	70
37	Department of Orthodontics, School of Dentistry, University of Texas Health Science Center, Houston, TX, United States	3	66
38	Department of Radiology, University of Michigan Medical School, Ann Arbor, MI, United States	3	65
39	Department of Cardiology, Taunton and Somerset NHS Trust, Taunton, Somerset, United Kingdom	2	63
40	Division of Infectious Diseases, Department of Medicine and Department of Cardiovascular Medicine, Mayo Clinic College of Medicine, Rochester, MN, United States	2	63
41	Department of Biomedical Engineering, College of Engineering, Ann Arbor, MI, United States	2	62
42	Department of Periodontics and Oral Medicine, University of Michigan School of Dentistry, Ann Arbor, MI, United States	2	62
43	School of Dentistry, College of Biomedical and Life Sciences, Cardiff University, Cardiff, United Kingdom	4	62
44	Department of Clinical Biology, Scientific Institute of Public Health, Brussels, Belgium	3	61
45	Department of Oral Surgery and Stomatology, School of Dental Medicine, University of Bern, Bern, Switzerland	2	61
46	Department of Oral and Maxillofacial Surgery, University Hospitals Leuven, Leuven, Belgium	3	60
47	Department of Orthodontics, Faculty of Odontology, Malmö University, Malmö, Sweden	2	59
48	Department of Dentistry, University of Alberta, Edmonton, AB, Canada	2	58
49	Department of Orthodontics, School of Dentistry, Chonnam National University, 33 Yongbong-ro, Buk-gu, Gwangju, 61186, South Korea	2	57
50	Department of Prosthetic Dentistry and Biomedical Materials Science, Hannover Medical School, Hannover, Germany	2	56

**Table 4:** Top 100 cited countries with documents published.

Country	Documents	Citations
United States	353	9468
United Kingdom	320	4982
Brazil	238	3215
Italy	116	2366
Germany	142	1830
Switzerland	89	1774
Belgium	60	1525
China	179	1484
Australia	70	1258
Netherlands	70	1199
Spain	58	1062
Sweden	75	1038
Japan	71	983
Turkey	55	946
Canada	34	778
South Korea	47	620
Saudi Arabia	92	596
India	83	468
Denmark	31	451
Thailand	21	415
Hong Kong	19	379
Jordan	21	372
Finland	32	341
France	24	338
Ireland	23	293
Iran	41	238
Malaysia	21	223
Egypt	43	208
Poland	15	203
Norway	18	191
Austria	13	186
Czech Republic	5	176
United Arab Emirates	16	176
Colombia	13	168
Portugal	19	162
Qatar	11	161
Slovenia	9	151
Hungary	19	150
Greece	8	147
Russian Federation	10	126
Israel	5	125
Taiwan	8	118
Iraq	16	114
Lithuania	11	112
Chile	10	106
Serbia	11	102

**Table 5:** Cooccurrence of author keywords with citations and total link strength.

Sr. no.	Keyword	Occurrences	Total link strength
1	Cone-beam computed tomography	90	207
2	Orthodontics	59	142
3	Dental caries	58	143
4	Dental implants	53	124
5	Covid-19	45	117
6	Radiography	40	108
7	Oral health	38	88
8	Cad/cam	36	84
9	Endodontics	36	84
10	Dental education	35	84
11	Diagnosis	34	96
12	Periodontitis	34	67
13	Cbct	33	64
14	Dentistry	31	93
15	Accuracy	28	70
16	Dental implant	26	77
17	Artificial intelligence	25	82
18	Epidemiology	23	68
19	Dental	22	61
20	Digital dentistry	22	53
21	Osteoporosis	22	49
22	Panoramic radiography	22	49
23	Radiology	21	56
24	Diagnostic imaging	20	58
25	Imaging	20	74
26	Intraoral scanner	20	48
27	Gingivitis	19	48
28	Oral hygiene	19	50
29	Periodontal disease	19	45
30	Deep learning	18	57
31	Social media	18	50
32	Digital impression	17	44
33	Mandible	17	44
34	Quality of life	17	40
35	Dental plaque	16	41
36	3d printing	15	37
37	Gingival recession	15	41
38	Malocclusion	15	29
39	Risk factors	15	40
40	Tooth wear	15	40
41	Dental students	14	34
42	Biofilm	13	22
43	Orthodontic treatment	13	33
44	Panoramic	13	44
45	Prosthodontics	13	35
46	Root canal treatment	13	25
47	Alveolar bone loss	12	34
48	Apical periodontitis	12	33
49	Caries detection	12	35
50	Dental radiography	12	27
51	Pandemic	12	34
52	Tooth	12	33
53	Cephalometry	11	19
54	Children	11	24
55	Cleft palate	11	29
56	Peri-implantitis	11	33
57	Prevalence	11	29
58	Remineralization	11	26
59	Reproducibility of results	11	26
60	Trueness	11	28
61	Cleft lip	10	26
62	Computer-aided design	10	23
63	Dentin	10	18
64	Dentists	10	21
65	Health services research	10	32
66	Magnetic resonance imaging	10	23
67	Maxillary sinus	10	26
68	Prevention	10	31

**Table 6:** Citations obtained by authors.

Sr. no.	Authors	Citations
1	Pulijala Y.; Ma M.; Pears M.; Peebles D.; Ayoub A.	158
2	Revilla-León M.; Jiang P.; Sadeghpour M.; Piedra-Cascón W.; Zandinejad A.; Özcan M.; Krishnamurthy V.R.	155
3	Thornhill M.H.; Dayer M.J.; Durkin M.J.; Lockhart P.B.; Baddour L.M.	63
4	Gallagher J.; Ashley P.; Petrie A.; Needleman I.	51
5	Spallek H.; Turner S.P.; Donate-Bartfield E.; Chambers D.; Mcandrew M.; Zarkowski P.; Karimbux N.	42
6	Abogazalah N.; Eckert G.J.; Ando M.	40
7	Sun X.; Bernabé E.; Liu X.; Gallagher J.E.; Zheng S.	29
8	Laukkanen E.; Vehkalahti M.M.; Kotiranta A.K.	25
9	Balasubramanian M.; Spencer A.J.; Short S.D.; Watkins K.; Chrisopoulos S.; Brennan D.S.	23
10	Coxon J.D.; Hosey M.-T.; Newton J.T.	12
11	Komabayashi T.; Ahn C.; Zhang S.; Zhu Q.; Spångberg L.S.W.	8
12	Buchanan G.D.; Gamieldien M.Y.; Fabris-Rotelli I.; Van Schoor A.; Uys A.	3
13	Wolfaardt J.F.; Brecht L.E.; Taft R.M.	3
14	Angerame D.; De Biasi M.; Franco V.; Bevilacqua L.; Castaldo A.	2

**Table 7:** Citations obtained by country-wise.

Sr. no.	Country	Documents	Citations	Total link strength
1	United States	353	3	16956
2	United Kingdom	320	946	13529
3	Brazil	238	3215	7936
4	China	179	1484	4404
5	Germany	142	338	8506
6	Italy	116	293	5908
7	Saudi Arabia	92	69	3985
8	Switzerland	89	1062	6573
9	India	83	379	3016
10	Sweden	75	620	5620
11	Japan	71	125	2767
12	Australia	70	1258	6804
13	Netherlands	70	55	4458
14	Belgium	60	1525	4252
15	Spain	58	151	5503
16	Turkey	55	118	1293
17	South Korea	47	41	1609
18	Egypt	43	6	2552
19	Iran	41	468	895
20	Canada	34	778	2219
21	Finland	32	12	1113
22	Denmark	31	451	2258
23	France	24	341	2442
24	Ireland	23	238	2158
25	Jordan	21	2366	1681
26	Malaysia	21	58	930
27	Thailand	21	1774	1043
28	Hong Kong	19	147	1439
29	Hungary	19	78	2591
30	Portugal	19	10	1177
31	Norway	18	43	788
32	Croatia	16	62	713
33	Iraq	16	47	825
34	United Arab Emirates	16	7	1851
35	Poland	15	31	889
36	Austria	13	186	1023
37	Colombia	13	168	1003
38	Indonesia	13	150	943
39	South Africa	12	70	688
40	Lithuania	11	35	1023
41	Qatar	11	203	997
42	Serbia	11	126	1621
43	Chile	10	106	2328
44	Russian Federation	10	161	1071
45	New Zealand	9	3	441
46	Slovenia	9	102	507
47	Greece	8	1830	1095
48	Singapore	8	596	553
49	Taiwan	8	1038	254
50	Kuwait	7	372	445
51	Mexico	7	112	199
52	Lebanon	6	53	292
53	Romania	6	162	288
54	Yemen	6	9468	575
55	Bosnia and Herzegovina	5	12	289
56	Czech Republic	5	176	690
57	Israel	5	114	195
58	Nigeria	5	1199	933
59	Pakistan	5	30	239
60	Latvia	4	5	776
61	Libyan Arab Jamahiriya	4	17	1100
62	Nepal	4	39	192
63	Peru	4	38	68
64	Sudan	4	61	199
65	Ukraine	4	415	6
66	Bulgaria	3	13	906
67	Ecuador	3	38	187
68	Morocco	3	223	282
69	Palestine	3	191	51
70	Slovakia	3	93	826
71	Syrian Arab Republic	3	21	231
72	Argentina	2	18	1601
73	Armenia	2	7	283
74	Bahrain	2	13	96
75	Bangladesh	2	6	79
76	Estonia	2	208	754
77	Kenya	2	983	42
78	Paraguay	2	55	180
79	Tunisia	2	31	29
80	Uruguay	2	176	92
81	Vietnam	2	4982	37

## Data Availability

All the associated data is included in the current analysis.
